# Insights Into the Gas‐Phase Structure and Internal Dynamics of Diphenylsilane: A Broadband Rotational Spectroscopy Study

**DOI:** 10.1002/cphc.202400790

**Published:** 2024-11-20

**Authors:** Gayatri Batra, Melanie Schnell

**Affiliations:** ^1^ Deutsches Elektronen-Synchrotron DESY Notkestr. 85 22607 Hamburg Germany; ^2^ Institute of Physical Chemistry Christian-Albrechts-Universität zu Kiel Max-Eyth-Str. 1 24118 Kiel Germany

**Keywords:** organosilicon molecules, gas-phase structure, tunnelling, rotational frequencies, microwave spectroscopy

## Abstract

The rotational spectrum of diphenylsilane was investigated using chirped‐pulse Fourier transform microwave spectroscopy in the frequency range of 2–8 GHz. The lowest energy structure of diphenylsilane has ***C*** 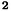 point group symmetry with the *C* 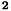 symmetry axis coinciding with the b ‐inertial axis of the molecule. Through the assignment of the main isotopologue as well as singly substituted heavy‐atom isotopologues, including 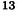 C, 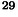 Si, and 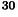 Si, we were able to obtain a comprehensive gas‐phase structure of diphenylsilane. The structure of diphenylsilane was compared with its oxygen analogue, diphenylether, including a discussion of the barrier height to the large‐amplitude motion of the phenyl rings. Furthermore, the structural comparison was extended to include a range of C−Si bond lengths and bond angles from other organosilicon molecules where the silicon atom is bonded to aliphatic and/or aromatic moieties.

## Graphical abstract

The structure of diphenylsilane could be determined from the rotational spectra of all its singly substituted heavy atom isotopologues. A comparison with its oxygen analogue diphenylether revealed some interesting differences in the internal dynamics as well as in the gas‐phase structure.

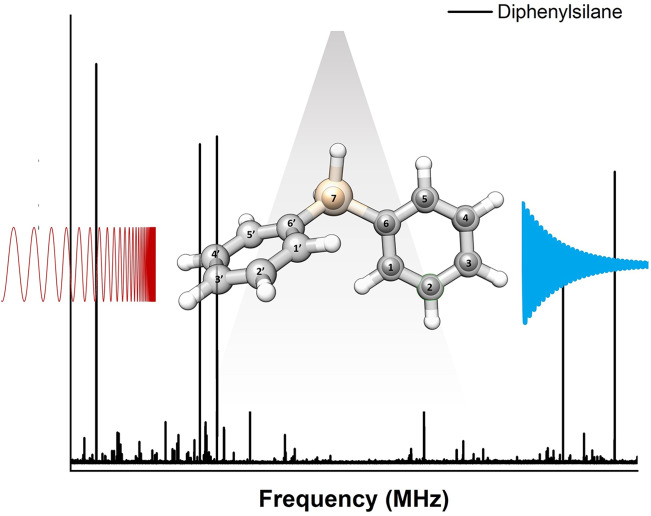

## Introduction

Silicon is the second most abundant element in the earth's crust accounting for around 27 % of the earth's crust by mass. It has an important role in modern‐day technology,[^1^, ^2^] medical applications,[^3^, ^4^] as well as in industry, ranging from semiconductors and biologically active compounds to silicones[^5^, ^6^] and other polymers.[^7^, ^8^, ^9^] Recently, silicon gained significant attention for its use in the form of organosilicon biomolecules. The inherent advantages of using silicon as a bioisostere of carbon make it an attractive option, such as its tetravalent nature akin to carbon, lack of toxicity,[^10^, ^11^] and higher lipophilicity[^3^, ^12^] compared to carbon. An interesting example involves the modification of the amino acid proline to create sila‐proline, which contains two methyl groups attached to silicon instead of a methylene carbon. This modification resulted in a 16‐fold increase in cellular uptake compared to proline.[^3^, ^4^]

In addition to the significant presence of silicon and its myriad of applications on earth, it is also the eighth most abundant element in the universe,^[13]^ widely distributed in dust particles and in many astronomical objects in various forms of silicates.^[14]^ In fact, small silicon‐bearing moieties are notably abundant in the interstellar medium (ISM) and play a key role in the chemical evolution of the ISM.[^15^, ^16^] The number of detected silicon‐containing molecular species in space constitutes a substantial portion, accounting for up to 7 % of the total number of molecular species identified in space so far.^[17]^ Organosilicon molecules like SiC, SiC_2_, *c*‐SiC_3_, SiC_4_, *etc*. are believed to be linked to the formation of silicon carbide dust grains in the outflow of circumstellar envelopes of carbon‐rich stars like IRC+10216.[^18^, ^19^, ^20^] Given the abundance of organosilicon molecules in space, silicon‐substituted analogs of known or postulated carbon moieties are likely targets for astronomical searches. Several silicon‐containing carbon chain radicals and their hydrogenated forms have been studied in silane‐acetylene electrical discharge experiments using Fourier transform microwave spectroscopy, motivated by the goal of detecting these species in the ISM.[^21^, ^22^, ^23^, ^24^, ^25^] As a next step in complexity, the detection of cyclic organosilicon molecules will provide valuable insights on the formation mechanisms of these molecules and contribute to our existing knowledge of silicon chemistry in space. To facilitate the potential detection of such molecules in space, laboratory data are required containing the spectroscopic signatures specific to that molecule.

Diphenylsilane is a complex organosilicon molecule with two phenyl groups attached to the Si center. We report its microwave spectroscopic analysis in the range of 2–8 GHz along with precise gas‐phase structure determination. In addition to the spectroscopic interest, investigating the structural properties of complex organosilicon molecules like diphenylsilane are the basis to its functional behavior, such as the chemical and biological activity. The ability of molecules to adjust their structures according to their chemical environment can be of significant importance for their chemical functioning, often resulting in large‐amplitude motions. For example, in the case of a similar molecule, diphenylether, the molecule undergoes significant structural adjustment to best accommodate the complexing partners like *tert*‐butyl alcohol.^[26]^ Such a structural flexibility can also manifest itself in internal dynamics, which often leads to characteristic line splittings in the rotational spectrum, and its analysis allows for the determination of the corresponding tunnelling pathways and associated barriers between the two equivalent forms. High‐resolution rotational spectroscopy complemented with quantum‐chemical calculations is ideally suited to investigate the structure, conformational flexibility, and internal dynamics of molecules.

In this work, we also investigate the tunnelling barrier height of diphenylsilane and compare it with the oxygen analogue, diphenylether.^[26]^ The comparison is then extended to the structural parameters as replacing oxygen with silicon in a molecule leads to an increase of the bond length from C−O to C−Si (due to the larger size of the silicon atom) and can result in structural modification and reactivity. Alongside this, the comprehensive rotational analysis of diphenylsilane and the generated line lists, rotational constants, and centrifugal distortion constants can aid future astronomical searches for diphenylsilane in the interstellar environments. Its detection will unveil an unexplored area of silicon‐substituted aromatic and cyclic molecules.

## Experimental and Theoretical Details

The liquid sample of diphenylsilane was purchased from Sigma‐Aldrich (97 % purity) and was used without any further purification. The broadband spectrum of diphenylsilane was obtained utilizing the COMPACT spectrometer.[^27^, ^28^, ^29^] The detailed information on the instrument and its working principle can be found in the mentioned references, and thus only a brief description is given here. The sample was brought into the gas phase via heating, followed by a supersonic expansion, where neon was used as a carrier gas at 200 kPa (2 bar) of backing pressure to generate a cold molecular jet. The sample was loaded into a modified pulsed nozzle (a modified Parker Series 9 valve with an orifice diameter of 1 mm, equipped with an internal heatable reservoir) and heated to 363 K to create sufficient vapour pressure. The resulting gas pulse (containing the sample molecules and the carrier gas) is expanded into the vacuum chamber by the pulsed nozzle operating at 8 Hz, where the molecular ensemble is polarised by the 4 μs chirp ranging from 2 to 8 GHz, resulting in a macroscopic dipole moment. The chirp is generated by an arbitrary waveform generator and amplified by a 300 W travelling wave tube amplifier prior to broadcasting it into the vacuum chamber using a horn antenna. We used eight consecutive excitation chirps per gas pulse resulting in an effective repetition rate of 64 Hz. Upon excitation by the chirped microwave pulses, the free induction decay (FID) of the macroscopic dipole moment of the molecular ensemble was recorded for 40 μs, with the resulting frequency resolution of 25 kHz and full width half maximum of 50–60 kHz. To obtain the final spectrum, 4.5 million FIDs were co‐added and Fourier transformed into the frequency domain.

The analysis of the experimental spectrum was facilitated by quantum‐chemical calculations using the ORCA 5.0 program package.[^30^, ^31^] The geometry optimizations followed by harmonic frequency calculations were performed at the B3LYP‐D3/aug‐cc‐pVTZ level of theory. The set of obtained theoretical rotational constants and electric dipole moments were used to simulate the rotational spectrum for the initial assignment process. In addition, Nudge Elastic Band (NEB) calculations were performed to estimate the transition barrier between the two equivalent minima.

For the initial spectral assignment, the calculated parameters for diphenylsilane were used as inputs to fit the experimental spectrum with PGOPHER.^[32]^ The fits were then refined with Pickett's SPCAT/SPFIT programs^[33]^ using Watson's *A*‐reduction Hamiltonian in the *I* ^*r*^ representation. The AABS package[^34^, ^35^] was used for displaying the predicted transitions and measuring the experimental frequencies. Finally, the EVAL program^[34]^ was employed for the calculation of bond lengths, bond angles, and dihedral angles using the Kraitchman's approach.

## Results and Discussion

**Figure 1:**
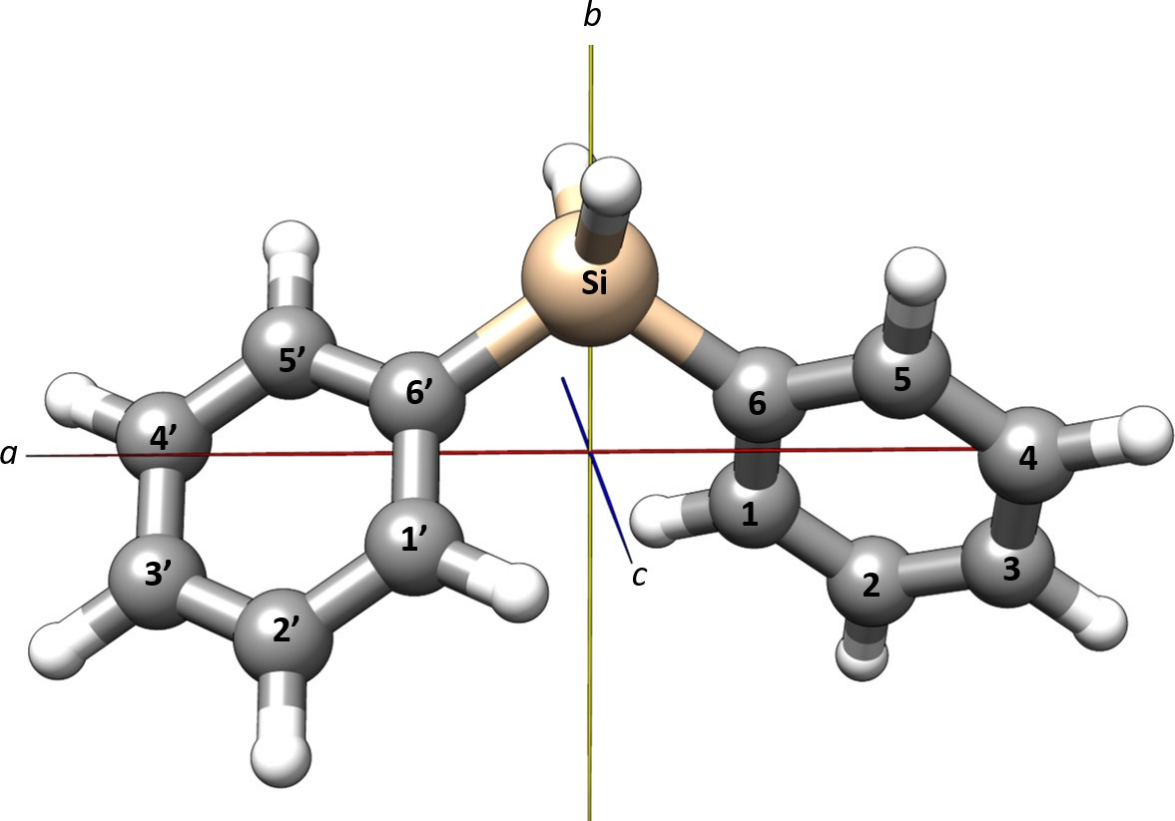
Molecular structure and principal inertial axes of diphenylsilane.

Diphenylsilane is a near‐prolate asymmetric top molecule with a value of Ray's asymmetry parameter of κ=-0.95 and a calculated electric dipole moment of 0.9 D along the *b*‐axis. The computed minimum energy structure of diphenylsilane (Figure 1) has a ***C***_**2**_ point group symmetry, with the *C*_2_ symmetry axis coinciding with the *b*‐inertial axis of the molecule. To find the most stable configuration of the molecule, a relaxed dihedral scan of the phenyl ring was performed in steps of 5° at the B3LYP‐D3/aug‐cc‐pVTZ level of theory. The relative energy of the molecule at different values of the dihedral angle is depicted in Figure S1 in the supplementary information. In the most stable configuration the two phenyl rings are arranged in such a way that the dihedral angle described by C1’−C6’−Si−C6 (see Figure 1 for numbering of atoms) corresponds to approximately 47° to minimize steric hindrance.

**Figure 2:**
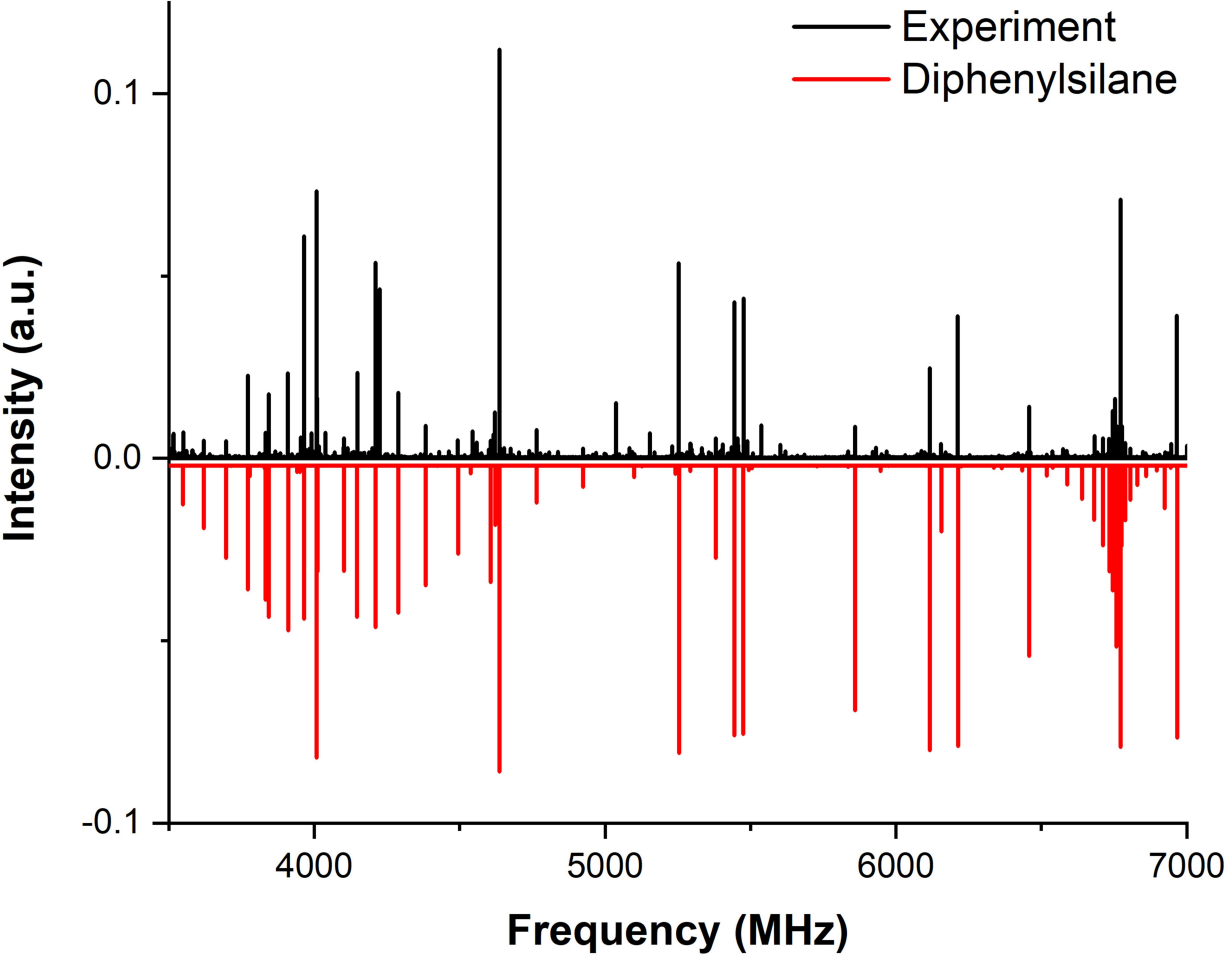
Part of the experimental rotational spectrum obtained for diphenylsilane in the frequency range of 2–8 GHz. The black trace represents the experimental spectrum, and the red trace corresponds to the simulated spectrum based on the fitted rotational constants (given in Table 1) and a rotational temperature of *T_rot_* =3 K.

A section of the high‐resolution broadband rotational spectrum of diphenylsilane is shown in Figure 2 with the experimental spectrum in the top trace (in black) and the simulated spectrum in the bottom trace (in red). The experimental rotational parameters obtained as a result of the fitting procedure are reported in Table 1 alongside the theoretically calculated ones. In total, 71 rotational transitions corresponding to the main isotopologue were assigned in the 2–8 GHz frequency range with a root‐mean‐square deviation of 9 kHz. The list of assigned transitions can be found in Table S1 in the Supplementary Information.

**Table 1:** Experimental and theoretical rotational parameters of the global minimum structure of diphenylsilane. The rotational transitions were fit using Watson's *A*‐reduction Hamiltonian in the *I* ^*r*^ representation. The theoretical rotational parameters were calculated at the B3LYP‐D3/aug‐cc‐pVTZ level of theory.

Rotational parameters	Experiment	Theory
*A* ^[a]^/MHz	1702.99149(88)	1722.37
*B*/MHz	366.69745(35)	367.30
*C*/MHz	335.80765(33)	333.48
Δ_*J*_^[b]^/kHz	0.0351(19)	
Δ_*JK*_/kHz	0.2100(70)	
Δ_*K*_/kHz	1.680(70)	
*μ_a_*/*μ_b_*/*μ_c_* ^[c]^/D		0/0.9/0
*κ*	−0.95	−0.95
*σ* ^[d]^/*kHz*	9	
No. of lines	71	

[a] A, B, and C are the rotational constants. [b] Δ_*J*_, Δ_*JK*_, and Δ_*K*_ are the quartic centrifugal distortion constants. [c] *μ_a_*/*μ_b_*/*μ_c_* are the electric dipole‐moment components. [d] Microwave root‐mean‐square deviation of the fit.

**Figure 3:**
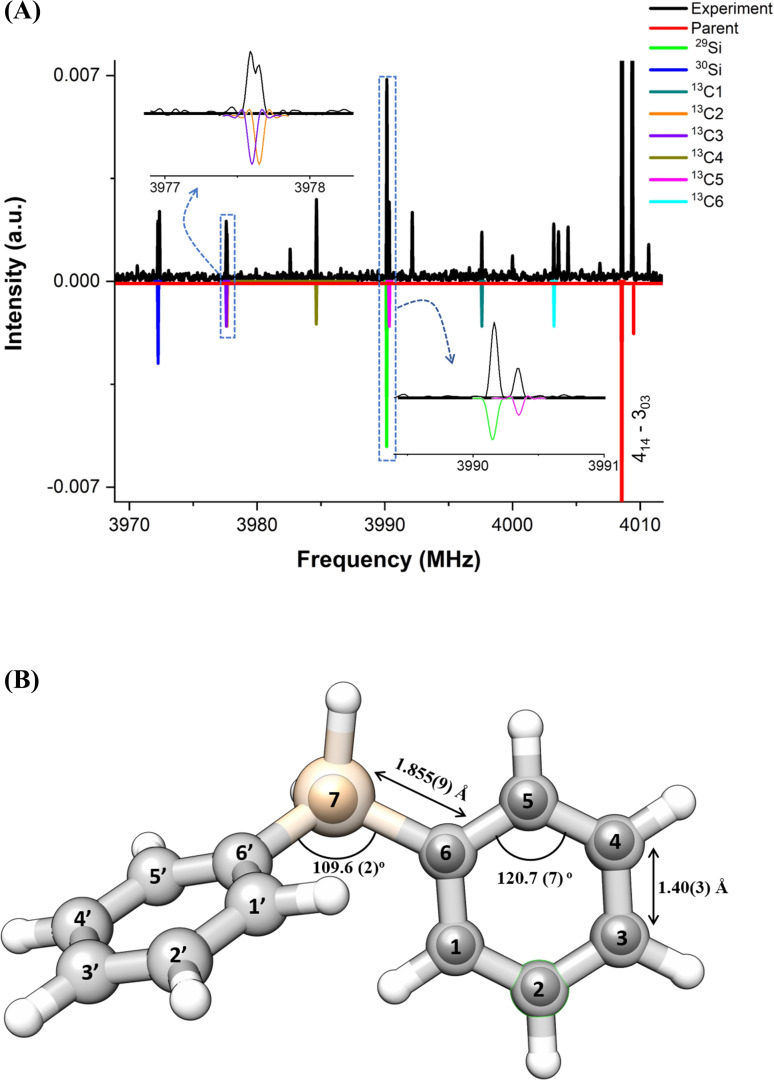
Panel A shows a section of the experimental spectrum highlighting the rotational transition $J_{K_{a},K_{c}}$ – $J_{K_{a}^{'},K_{c}^{'}}^{'}$ $=4_{14}-3_{03}$ for the main isotopologue (in red) and for all the observed singly substituted rare isotopologues as given in the legend. The sections where the rotational transitions arising from different isotopologues are close to each other are shown in the zoom‐in of the spectrum. Panel B shows the experimental gas‐phase structure of diphenylsilane. The inner bold spheres represent the r_*s*_ positions of the atoms, whereas the partially transparent backbone gives the theoretical r_*e*_ structure (at B3LYP‐D3/aug‐cc‐pVTZ level of theory).

To our advantage, the signal‐to‐noise ratio of the experimental spectrum was sufficient to observe the rotational spectra of six singly substituted ^13^C isotopologues and two singly substituted Si isotopologues, with ^29^Si and ^30^Si, in their natural abundances of 1.1 %, 4.6 %, and 3.1 % for ^13^C, ^29^Si, and ^30^Si, respectively. Due to the overall ***C***_***2***_ symmetry of the molecule, the rotational transitions arising from ^13^C isotopologues exhibit double the intensity (~2.2 %) as compared to the expected natural abundance of 1.1 %. This effect is further highlighted in Figure 3, where a section of the experimental spectrum is shown against the simulated spectra of the singly substituted ^13^C, ^29^Si, and ^30^Si isotopologues, for the rotational transition $J_{K_{a},K_{c}}-J_{K_{a}^{'},K_{c}^{'}}^{'}=4_{14}-3_{03}$ . The merged transitions are shown separately in the zoom‐in of Figure 3(A) for better visualization of the observed rotational transitions. The fitted rotational and quartic centrifugal distortion constants of the singly substituted heavy‐atom isotopologues containing ^13^C, ^29^Si, and ^30^Si are summarized in Table S2, and the list of their measured frequencies can be found in Tables S4–S11 in the supplementary information.

The set of rotational constants obtained from the fits of these isotopologues allow for the determination of the individual atom positions with respect to the center of mass of the molecule. This results in an accurate determination of the experimental structure of the molecule in the gas phase, which can then be compared to the theoretical structure (r_*e*_) based on quantum‐chemical calculations.

In order to experimentally determine the gas‐phase structure of a molecule, different approaches can be employed. In this work, Kraitchman's equations were used to obtain the substitution structure, r_*s*_.^[36]^ Kraitchman's substitution method exploits changes in the moments of inertia upon single isotopic substitution, either in natural abundance or as an enriched sample. This approach allows for determination of the coordinates of each isotopically substituted atom in the principal inertial axis frame (the signs of the coordinates can be assigned from the theoretical or effective structure coordinates). The various structural parameters were derived from a r_*s*_ structure determination using the EVAL program.^[34]^ All the relevant bond distances, bond angles, and dihedral angles for the gas‐phase structure of diphenylsilane determined in this work are given in Table S3 in the supplementary information. Figure 3(B) shows the overlay of the experimentally obtained r_*s*_ structure (in bold spheres) and the theoretical structure r_*e*_ obtained at the B3LYP‐D3/aug‐cc‐pVTZ level of theory (in partially transparent backbone).

### Structure Comparison

The structure of diphenylsilane can be compared with that of diphenylether^[26]^ – the oxygen analogue of diphenylsilane. It is important to note that different methods were employed to determine the structural parameters from the experimental data for both molecules, r_*s*_ for diphenylsilane and r_0_ (using a least‐squares fitting method^[42]^) for diphenylether. The r_*s*_ structure is closer to the equilibrium structure, r_*e*_, than the r_0_ structure, nevertheless both methods provide reliable structural information. In comparison, the C−Si bond length is found to be evidently longer than that of C−O with values of 1.855(9) Å for C−Si and 1.406(35) Å for C−O. The longer bond length observed for diphenylsilane in comparison to diphenylether can be explained by the larger radius of the Si atom (1.17 Å)^[43]^ with respect to the O atom (0.66 Å).^[43]^ Similarly, the dihedral angle described by C6’−Si−C6−C1 in diphenylsilane is found to be $47{\left(1\right)}^{\circ }$ compared to $43{\left(2\right)}^{\circ }$ for diphenylether. The bond angle between C−Si−C is $109.6{\left(2\right)}^{\circ }$ , which demonstrates the perfect tetrahedral arrangement corresponding to the *sp* ^3^ hybridization of the silicon atom. While for the C−O−C angle in diphenylether, amounting to $116{\left(3\right)}^{\circ }$ , the steric hindrance created by the two phenyl groups attached to an oxygen atom might cause the deviation (increase) in the bond angle due to the smaller size of O as compared to Si.

In addition to diphenylether, the structural comparison was also extended to other organosilicon molecules, for comparing the corresponding Si−C bond lengths to the experimental one for diphenylsilane, as shown in Table 2. The list includes two rhomboidal isomers of cyclic silicon tricarbide (*c*‐SiC_3_) and their corresponding transannular and peripheral Si−C bonds,[^37^, ^38^] where Si is included in the cyclic structure of the molecule, trimethylsilyl chloride containing three methyl groups attached to Si,^[39]^ phenylsilane^[44]^ and methyl phenylsilane,^[41]^ where Si is attached to an aromatic ring. Note that no rotational spectroscopy data is available for the gas‐phase structure determination of phenylsilane and methyl phenylsilane. The Si−C bond lengths reported in Table 2 for methyl phenylsilane and phenylsilane were obtained computationally at the MP2/aug‐cc‐pVTZ^[41]^ and at the MP2/6‐31G*^[40]^ level of theory, respectively. For phenylsilane, the Si−C bond length is also reported from the gas‐phase electron diffraction study by Portalone *et al*.^[40]^

**Table 2:** A comparison of Si−C bond lengths of different organosilicon molecules, in Å, with that of diphenylsilane determined experimentally and from structure optimisation at the B3LYP‐D3/aug‐cc‐pVTZ level of theory.

Molecule	Si−C bond length (Å)	Reference
(C_6_H_5_)_2_SiH_2_ (r_*s*_)	1.855(9)	This work
(C_6_H_5_)_2_SiH_2_ (r_*e*_)	1.876	B3LYP‐D3/ aug‐cc‐pVTZ
Ground state‐SiC_3_ (r_0_)	1.834(2)	[37]
Oblate ring‐SiC_3_ (r_0_)	1.893(1)^[a]^/2.022(1)^[b]^	[38]
(CH_3_)_3_SiCl (r_0_)	1.861(1)	[39]
C_6_H_5_SiH_3_ (r_*e*_)	1.872	[40], MP2/6‐31G*
C_6_H_5_SiH_3_ (ED)^[e]^	1.870(4)	[40]
C_6_H_5_SiH_2_CH_3_ (r_*e*_)	1.877^[c]^/1.879^[d]^	[41], MP2/ aug‐cc‐pVTZ

[a, b] Transannular/peripheral Si−C bond length. [c, d] Aromatic/aliphatic Si−C bond length. [e] Results from electron diffraction study.

As can be seen from Table 2, the longest Si−C bond lengths are reported for the second rhomboidal isomer of SiC_3_, referred to as oblate ring structure of SiC_3_ (1.893(1) and 2.022(1) Å) for transannular and peripheral Si−C bonds, respectively. This is because in oblate ring‐SiC_3_, silicon is a part of a strained four‐membered ring and is connected to three carbon atoms with single bonds within the ring. In order to reduce the ring strain, the silicon atom is pushed away from the ring, giving rise to longer Si−C bond lengths as compared to other entries in Table 2. Whereas, in the ground state rhomboidal isomer of SiC_3_, the Si atom is connected to two carbon atoms with single bonds, while still being a part of the four‐membered ring, resulting in lower strain and consequently shorter Si−C bond lengths with respect to the oblate ring structure of SiC_3_. One can also compare the Si−C bond lengths when the silicon atom is connected to the aromatic or aliphatic carbon atoms as in the case of diphenylsilane and trimethylsilyl chloride, respectively. The Si−C bond length in diphenylsilane is slightly shorter (1.855(9) Å) than that in trimethylsilyl chloride (1.861(1) Å), since Si is connected to an aliphatic carbon in the case of trimethylsilyl chloride, giving rise to a longer Si−C bond length.

### Comparison of Barrier Heights

To understand the internal rotation dynamics of diphenylsilane, we compared our study with its oxygen analogue, diphenylether. In diphenylether, the two phenyl rings are attached to the oxygen atom via C−O single bonds, these rings can rotate around their respective C−O bonds. This rotation can be hindered by a finite barrier, allowing the phenyl groups to transition between equivalent minima. This was exhibited in the microwave spectrum of diphenylether, where the splittings of rotational transitions due to the tunnelling motion of the phenyl rings were observed.^[26]^ The barrier height to internal rotation along the C−O bond was reported to be approximately 100 cm^−1^ (1.2 kJ/mol) at the B3LYP‐D3/def2‐TZVP level of theory. This barrier caused each rotational transition to split into three components, with an average splitting of about 240 kHz across the spectrum. However, in our experiment with diphenylsilane, no such splittings in the rotational transitions were observed. To provide some insight into the difference in the internal motion of phenyl groups around the Si or O center, we performed NEB calculations at the B3LYP‐D3(BJ)/def2‐TZVP level of theory to identify and optimize the transition state (identified by the presence of a single imaginary frequency) involved in the motion followed by single point energy calculations at the DLPNO(TightPNO)‐CCSD(T1) level of theory for the equilibrium structure and the transition state.

We also extended the comparison to diphenylmethane. To the best of our knowledge, no rotational spectroscopy study has been conducted on diphenylmethane to date, and therefore, no experimental data on the tunnelling barrier is available. The theoretical barrier height of 133 cm^−1^, calculated at the same level of theory as for diphenylsilane and diphenylether, is higher than for diphenylsilane and diphenylether due to steric hindrance so that no splitting in the rotational transitions can be expected.

**Figure 4:**
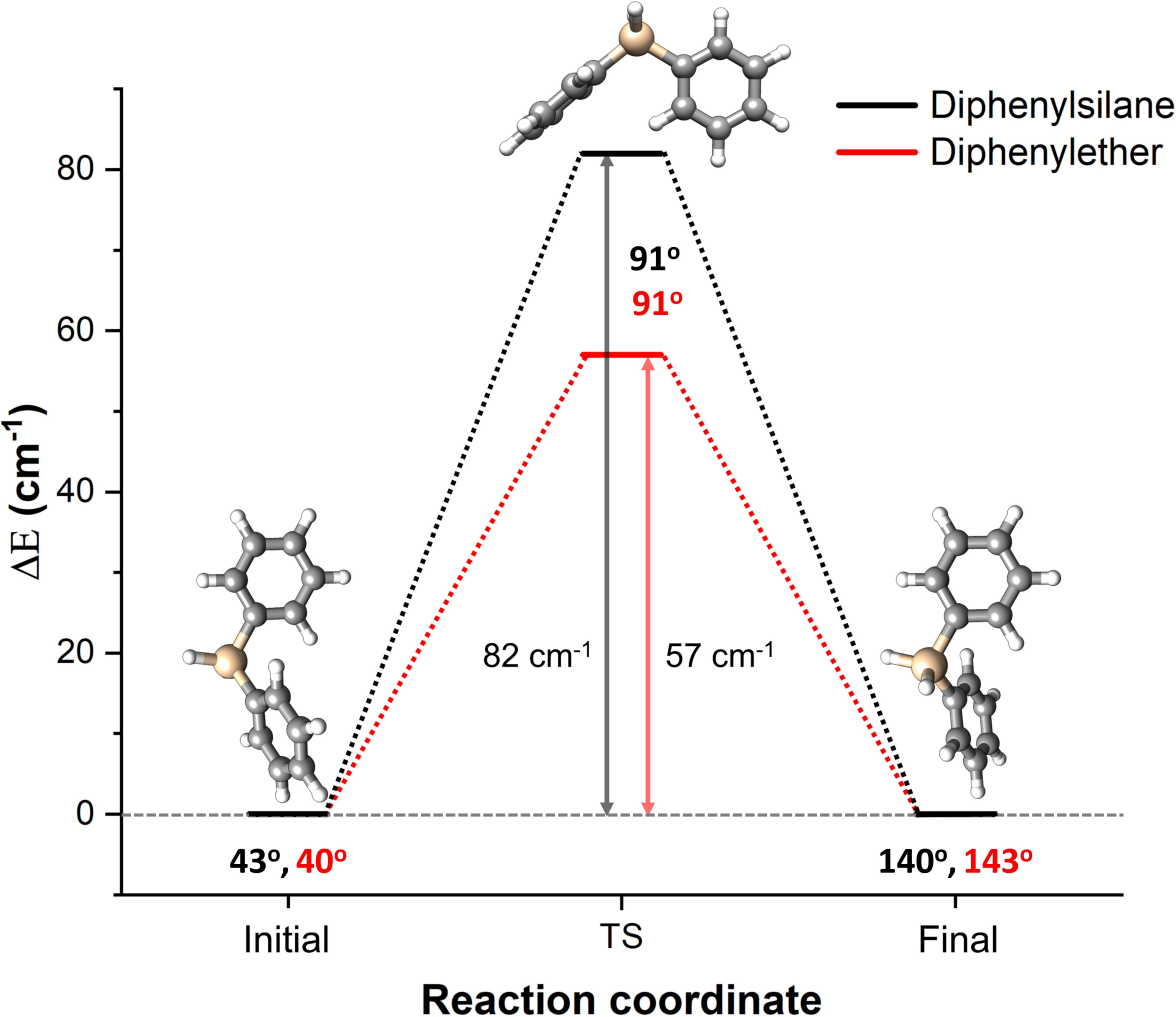
Theoretical analysis of the barrier height to the internal motion of the phenyl rings calculated at the DLPNO(TightPNO)‐CCSD(T1)/cc‐pVTZ//B3LYP‐D3(BJ)/def2‐TZVP level of theory. The *x*‐axis corresponds to the reaction coordinate, where the molecule goes from the equilibrium structure to the transition state structure, shown by the change in dihedral angle 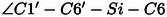 (given under the respective structure). The *y*‐axis corresponds to the energy difference between the equilibrium structure and the transition state structure. The calculated energy difference and the dihedral angles are shown for diphenylether in red and for diphenylsilane in black.

The observed barrier heights to the motion of the phenyl rings at the DLPNO(TightPNO)‐CCSD(T1)/cc‐pVTZ//B3LYP‐D3(BJ)/def2‐TZVP levels of theory for both diphenylsilane and diphenylether are depicted in black and red curves in Figure 4, respectively. The slightly higher barrier to internal rotation in diphenylsilane (82 cm^−1^) compared to diphenylether (57 cm^−1^), though probably within the accuracy of the quantum‐chemical methods, could explain the absence of detectable splittings of the rotational transitions in the microwave spectrum of diphenylsilane. To further validate these theoretical results, we performed additional single‐point energy calculations based on geometries optimized at the B3LYP‐D3(BJ)/def2‐TZVP level of theory, using various additional methods namely CCSD(T), CC2, and MP2. The results and further information on the respective basis sets are given in Table S12. All the above‐mentioned methods indicate a slightly higher barrier height for diphenylsilane compared to diphenylether.

## Conclusions

Diphenylsilane is a complex organosilicon molecule with 13 heavy atoms. The broadband rotational spectrum of diphenylsilane is reported in the frequency range of 2–8 GHz. The comprehensive rotational spectroscopic analysis for this mole‐cule sheds light on its gas‐phase structure and its internal dynamics, which shows that it is rather rigid under the cold conditions of a molecular jet and does not show any splitting in the rotational transitions characteristic of large‐amplitude motion. The rotational spectra of the parent isotopologue of diphenylsilane and all its singly substituted heavy‐atom isotopologues, namely ^13^C, ^29^Si, and ^30^Si, have been assigned with the help of theoretical calculations performed at the B3LYP‐D3/aug‐cc‐pVTZ level of theory.

The gas‐phase structure of diphenylsilane was determined using the Kraitchman's equations employed in the r_*s*_ method. A good agreement was observed between the theoretical atomic coordinates and the experimentally determined ones. The structural parameters like bond lengths, bond angles, and dihedral angles of diphenylsilane were compared with the structure of its oxygen analogue, diphenylether. This allowed for the identification of changes in the bond angles and bond lengths when the hetero atom is changed from O to Si. This structural comparison was also extended to other silicon containing molecules like rhomboidal silicon tricarbide, trimethylsilyl chloride, phenylsilane, and methyl phenylsilane.

The calculated barrier height for phenyl internal rotation around the Si atom of diphenylsilane was compared with its oxygen analogue, diphenylether.^[26]^ Diphenylsilane was found to have a slightly higher barrier to phenyl internal rotation than that of diphenylether, which could explain the absence of resolvable splittings observed experimentally. Given the size difference between the atomic radii of Si (1.17 Å) and O (0.66 Å), one would expect a smaller barrier to the internal rotation of the phenyl groups for diphenylsilane than that of diphenylether, as the Si−C (1.855±0.009 Å) bond length is considerably longer than that of O−C (1.406±0.035 Å). However, this was not observed in our experiment.

As an outlook, the broadband rotational spectroscopy study of diphenylsilane can be potentially extended to high frequency regions, such as the 75–110 GHz range, which overlaps with ALMA Band 3.^[45]^ Such measurements can provide accurate rest frequencies for detecting complex organosilicon molecules like diphenylsilane especially in the regions where other silicon‐containing molecules have been previously detected in high abundances, for example, in the carbon‐rich star IRC+10216.^[46]^ The detection of complex organosilicon molecules will enable us to understand the silicon chemistry taking place in the ISM and its existing chemical complexity.

## Conflict of Interests

The authors declare that they have no known competing financial interests or personal relationships that could have appeared to influence the work reported in this paper.

1

## Supporting information

As a service to our authors and readers, this journal provides supporting information supplied by the authors. Such materials are peer reviewed and may be re‐organized for online delivery, but are not copy‐edited or typeset. Technical support issues arising from supporting information (other than missing files) should be addressed to the authors.

Supporting Information

## Data Availability

The data that support the findings of this study are available in the supplementary material of this article.
