# Supplementary material for: Insights Into the Gas‐Phase Structure and Internal Dynamics of Diphenylsilane: A Broadband Rotational Spectroscopy Study
Source: Chemphyschem. 2024 Nov 20;26(2):e202400790. doi: 10.1002/cphc.202400790 (PMC11733410; doi:10.1002/cphc.202400790)
Supplement: Supplementary file 1 — Supporting Information [file CPHC-26-e202400790-s001.pdf]

ChemPhysChem

Supporting Information

Insights Into the Gas-Phase Structure and Internal Dynamics of Diphenylsilane: A Broadband Rotational Spectroscopy Study

Gayatri Batra and Melanie Schnell*

Supplementary Information

Insights into the gas phase structure and internal dynamics of diphenylsilane: A broadband rotational spectroscopy study

Gayatri Batra[a,b] and Melanie Schnell[a,b]*

October 11, 2024

[a] *Deutsches Elektronen-Synchrotron DESY, Notkestr. 85, 22607 Hamburg, Germany.*

[b] *Institute of Physical Chemistry, Max-Eyth-Str. 1, Christian-Albrechts-Universität zu Kiel, 24118 Kiel, Germany.*

[*] *Corresponding author, Email: melanie.schnell@desy.de*

Contents

S1 List of measured rotational frequencies for the main isotopologue. 4

S2 The experimental rotational constants for the heavy-atom isotopologues of diphenylsilane. 8

S3 Structural parameters of diphenylsilane derived using the r_s method. 10

S4 List of measured rotational frequencies for heavy-atom rare isotopologues. 11

S5 Comparison of barrier heights at different levels of theory. 28

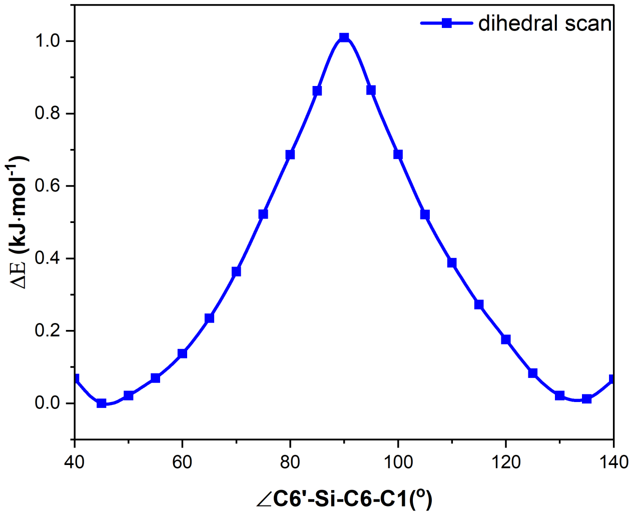

Figure S1: Relaxed dihedral scan of the phenyl rings in diphenylsilane in steps of 5°. The scan was performed for the dihedral angle C1′-C6′-Si-C6 (see Figure 1 in this manuscript), at B3LYP-D3/aug-cc-pVTZ level of theory.

S1.0 List of measured rotational frequencies for the main isotopologue.

Table S1: List of assigned rotational transitions of the main isotopologue of diphenylsilane ($C_{12}H_{10}Si$) in 2-8 GHz frequency range.

J	K_a	K_c	J'	K'_a	K'_c	**Observed Freq. (MHz)**	**Residuals (MHz)**
2	2	1	1	1	0	5444.7475	-0.004
2	2	1	2	1	2	4101.5207	-0.002
3	1	3	2	0	2	3366.7700	-0.008
3	2	1	2	1	2	6211.6785	0.004
8	1	8	7	0	7	6457.4680	0.010
2	1	2	1	0	1	2710.4089	-0.001
4	1	4	3	0	3	4008.5690	-0.002
5	1	5	4	0	4	4636.8617	-0.007
6	1	6	5	0	5	5253.1386	-0.012
7	1	7	6	0	6	5859.2899	0.008
3	2	2	2	1	1	6116.3477	-0.012
4	2	3	3	1	2	6772.4375	-0.004
5	2	4	4	1	3	7413.0496	-0.002
10	1	10	9	0	9	7639.8698	0.009
9	1	9	8	0	8	7050.1298	0.005

J	K_a	K_c	J'	K'_a	K'_c	**Observed Freq. (MHz)**	**Residuals (MHz)**
9	0	9	8	1	8	5380.9992	0.002
10	0	10	9	1	9	6154.7770	0.003
12	0	12	11	1	11	7688.9019	-0.003
8	0	8	7	1	7	4605.7971	-0.013
7	0	7	6	1	6	3831.8359	0.004
6	0	6	5	1	5	3061.7444	0.003
14	2	12	14	1	13	3400.5998	-0.031
12	2	10	12	1	11	3403.1369	-0.004
11	2	9	11	1	10	3436.5584	-0.007
10	2	8	10	1	9	3486.6293	0.001
9	2	7	9	1	8	3549.3488	0.004
8	2	6	8	1	7	3620.5488	-0.006
7	2	5	7	1	6	3696.0680	-0.006
6	2	4	6	1	5	3771.8581	-0.003
5	2	3	5	1	4	3844.1618	-0.006
4	2	2	4	1	3	3909.6536	-0.004
3	2	1	3	1	2	3965.4886	-0.006
2	2	0	2	1	1	4009.3766	-0.006
2	2	1	2	1	2	4101.5210	-0.002
3	2	2	3	1	3	4148.1866	0.005
4	2	3	4	1	4	4210.5876	0.001

J	K_a	K_c	J'	K'_a	K'_c	**Observed Freq. (MHz)**	**Residuals (MHz)**
5	2	4	5	1	5	4288.8905	0.005
6	2	5	6	1	6	4383.2503	0.006
7	2	6	7	1	7	4493.8400	0.008
8	2	7	8	1	8	4620.8173	0.010
9	2	8	9	1	9	4764.3123	0.009
10	2	9	10	1	10	4924.4171	0.006
11	2	10	11	1	11	5101.1821	0.017
2	2	1	1	1	0	5444.7483	-0.003
2	2	0	1	1	1	5476.1679	-0.002
4	2	2	3	1	3	6965.7100	0.005
5	2	3	4	1	4	7740.3845	0.005
7	3	5	7	2	6	6776.5506	0.004
8	3	6	8	2	7	6788.6450	-0.003
6	3	4	6	2	5	6768.3800	-0.017
4	3	2	4	2	3	6760.3460	0.000
3	3	1	3	2	2	6758.8915	-0.009
3	3	0	3	2	1	6756.2532	-0.008
4	3	1	4	2	2	6752.4518	-0.004
5	3	2	5	2	3	6744.9541	-0.007
6	3	3	6	2	4	6732.0465	0.000
7	3	4	7	2	5	6711.7801	0.013

J	K_a	K_c	J'	K'_a	K'_c	**Observed Freq. (MHz)**	**Residuals (MHz)**
8	3	5	8	2	6	6682.1018	0.011
9	1	8	8	2	7	2933.4306	-0.009
8	4	4	9	3	7	3125.9643	-0.002
4	3	2	5	2	3	3230.5445	-0.008
4	3	1	5	2	4	3249.0586	-0.004
13	2	11	13	1	12	3390.0359	-0.014
11	3	8	11	2	9	6518.5690	0.025
10	3	7	10	2	8	6586.9674	0.024
5	3	3	5	2	4	6763.2992	0.026
9	3	7	9	2	8	6805.7072	0.001
11	3	9	11	2	10	6858.9747	-0.011
12	3	10	12	2	11	6897.4312	0.000
11	0	11	10	1	10	6924.7405	-0.007
13	3	11	13	2	12	6945.2159	-0.022

S2.0 The experimental rotational constants for the heavy-atom isotopologues of diphenylsilane.

Table S2: Experimental rotational constants obtained for the observed isotopologues of diphenylsilane. Parameters are obtained by using the SPFIT program.

**Rotational parameters**	^{29}**Si**	^{30}**Si**	^{13}**C1**	^{13}**C2**	^{13}**C3**	^{13}**C4**	^{13}**C5**	^{13}**C6**
**A^{a}/MHz**	1689.10071(27)	1675.61231(31)	1696.33488(53)	1686.74297(35)	1696.23332(43)	1700.45898(44)	1696.2776(11)	1701.53655(70)
**B/MHz**	366.70091(19)	366.70388(21)	365.81859(32)	364.475180(21)	362.78387(25)	362.76939(36)	364.70283(50)	366.08671(36)
**C/MHz**	335.27005(13)	334.74088(14)	335.14622(19)	333.688440(17)	332.27089(19)	332.60598(19)	334.10348(46)	335.24340(26)
Δ_J^b**/kHz**	[0.035078254]	[0.035078254]	[0.035078254]	[0.035078254]	[0.035078254]	[0.035078254]	[0.035078254]	[0.035078254]
Δ_{JK}**/kHz**	[0.209956496]	[0.209956496]	[0.209956496]	[0.209956496]	[0.209956496]	[0.209956496]	[0.209956496]	[0.209956496]
Δ_K**/kHz**	[1.680165342]	[1.680165342]	[1.680165342]	[1.680165342]	[1.680165342]	[1.680165342]	[1.680165342]	[1.680165342]
σ^c**/kHz**	5	5	5	5	6	6	5	8
**No. of lines**	40	31	20	28	25	24	25	25

[a] A, B, and C are the rotational constants.

[b] Δ_J, Δ_{JK}, Δ_K are the quartic centrifugal distortion constants.

[c] Microwave root-mean-square deviation of the fit.

S3.0 Structural parameters of diphenylsilane derived using the r_S method.

Table S3: The relevant bond lengths, bond angles, and dihedral angles for the gas-phase structure of diphenylsilane. The structural parameters were calculated using the r_S method.

**Bond lengths (Å)**	
C1-C2	1.389 (2)
C2-C3	1.407 (8)
C3-C4	1.397 (34)
C4-C5	1.385 (22)
C5-C6	1.443 (10)
C6-Si	1.856 (10)
**Bond angles (°)**	
C1-C6-C5	117.1(2)
Si-C6-C5	120.7(7)
C6-Si-C6′	109.6(2)
**Dihedral angle (°)**	
C6′-Si-C6-C1	47(2)

S4.0 List of measured rotational frequencies for heavy-atom rare isotopologues.

Table S4: List of assigned rotational transitions of ^{29}Si diphenylsilane in the 2-8 GHz frequency range.

J	K_a	K_c	J'	K'_a	K'_c	**Observed Freq. (MHz)**	**Residuals (MHz)**
2	1	2	1	0	1	2694.9040	-0.0028
2	2	0	1	1	1	5434.5232	-0.0026
2	2	1	1	1	0	5402.5333	-0.0079
3	1	3	2	0	2	3349.9427	0.0045
3	2	2	3	1	3	4108.9541	0.0056
4	1	4	3	0	3	3990.1631	-0.0017
4	2	2	4	1	3	3866.4826	-0.0086
4	2	3	4	1	4	4172.4614	-0.0024
5	0	5	4	1	4	2310.9990	-0.0008
5	1	5	4	0	4	4616.7139	-0.0012
5	2	3	4	1	4	7700.3833	0.0067
5	2	4	5	1	5	4252.1623	-0.0011
6	1	6	5	0	5	5231.1334	-0.0051
6	2	5	6	1	6	4348.2262	0.0077
7	0	7	6	1	6	3844.7555	-0.0038
7	2	6	7	1	7	4460.8005	-0.0033

J	K_a	K_c	J'	K'_a	K'_c	**Observed Freq. (MHz)**	**Residuals (MHz)**
8	1	8	7	0	7	6431.7350	0.0018
4	2	3	3	1	2	6727.7995	-0.0075
3	2	2	2	1	1	6073.0715	-0.0030
7	1	7	6	0	6	5835.3811	-0.0001
5	2	4	4	1	3	7366.7990	0.0026
6	2	5	5	1	4	7990.1658	0.0002
9	1	9	8	0	8	7022.7377	0.0012
8	2	6	8	1	7	3575.4410	0.0003
6	2	4	6	1	5	3727.1852	0.0004
3	2	1	3	1	2	3923.1298	-0.0068
2	2	0	2	1	1	3967.7197	-0.0044
2	2	1	2	1	2	4061.4550	-0.0080
8	2	7	8	1	8	4590.0729	-0.0083
6	3	3	6	2	4	6662.7430	0.0106
5	3	2	5	2	3	6676.2231	-0.0019
4	3	1	4	2	2	6684.0576	-0.0007
3	3	0	3	2	1	6688.0296	-0.0067
3	3	1	3	2	2	6690.7964	-0.0010
4	3	2	4	2	3	6692.3135	0.0046
5	3	3	5	2	4	6695.3755	0.0069
6	3	4	6	2	5	6700.7248	0.0011

J	K_a	K_c	J'	K'_a	K'_c	**Observed Freq. (MHz)**	**Residuals (MHz)**
7	3	5	7	2	6	6709.2412	0.0032
3	2	1	2	1	2	6170.1339	-0.0003
4	2	2	3	1	3	6924.6928	0.0135

Table S5: List of assigned rotational transitions of ^{30}Si diphenylsilane in the 2-8 GHz frequency range.

J	K_a	K_c	J'	K'_a	K'_c	**Observed Freq. (MHz)**	**Residuals (MHz)**
2	1	2	1	0	1	2679.8307	-0.0002
2	2	0	1	1	1	5394.0875	-0.0006
2	2	1	1	1	0	5361.5423	-0.0046
2	2	1	2	1	2	4022.5854	0.0001
3	1	3	2	0	2	3333.5410	-0.0063
3	2	1	2	1	2	6129.7955	-0.0054
3	2	2	2	1	1	6031.0226	0.0008
3	2	2	3	1	3	4070.8895	0.0053
4	1	4	3	0	3	3972.2326	-0.0012
4	2	3	4	1	4	4135.4916	-0.0010
5	1	5	4	0	4	4597.0629	-0.0067
5	2	3	4	1	4	7661.5779	0.0031
7	1	7	6	0	6	5812.0791	0.0055
8	1	8	7	0	7	6406.6610	0.0051
4	2	3	3	1	2	6684.4247	-0.0016
9	1	9	8	0	8	6996.0621	0.0053
5	2	4	4	1	3	7321.8161	-0.0051
4	2	2	3	1	3	6884.8570	-0.0022
6	3	3	6	2	4	6595.3986	0.0022

J	K_a	K_c	J'	K'_a	K'_c	**Observed Freq. (MHz)**	**Residuals (MHz)**
7	3	4	7	2	5	6573.3254	-0.0037
3	3	1	3	2	2	6624.6860	-0.0001
4	3	2	4	2	3	6626.2586	-0.0060
5	3	3	5	2	4	6629.4526	-0.0065
6	3	4	6	2	5	6635.0592	0.0111
10	2	9	10	1	10	4874.9427	0.0018
5	2	4	5	1	5	4216.5829	0.0120
2	2	0	2	1	1	3927.2749	0.0003
3	2	1	3	1	2	3881.9945	-0.0084
4	2	2	4	1	3	3824.5787	0.0090
7	2	5	7	1	6	3607.2280	0.0000
6	0	6	5	1	5	3087.2297	-0.0034

Table S6: List of assigned rotational transitions of ^{13}C1 diphenylsilane in the 2-8 GHz frequency range.

J	K_a	K_c	J'	K'_a	K'_c	**Observed Freq. (MHz)**	**Residuals (MHz)**
2	2	0	1	1	1	5455.3177	0.0012
2	2	1	1	1	0	5424.1141	-0.0058
3	2	1	2	1	2	6189.0430	0.0003
3	2	2	2	1	1	6094.4073	0.0018
3	2	2	3	1	3	4129.8734	0.0068
4	1	4	3	0	3	3997.5972	-0.0012
4	2	3	3	1	2	6749.2762	0.0024
5	0	5	4	1	4	2295.3941	0.0072
5	1	5	4	0	4	4624.8655	-0.0037
5	2	4	4	1	3	7388.7899	0.0108
6	2	5	6	1	6	4363.2578	-0.0080
7	1	7	6	0	6	5845.4431	0.0049
2	1	2	1	0	1	2701.7659	-0.0036
3	1	3	2	0	2	3356.9183	-0.0029
6	1	6	5	0	5	5240.1960	-0.0048
8	1	8	7	0	7	6442.7492	-0.0079
9	2	7	9	1	8	3534.7525	-0.0041
5	2	3	5	1	4	3827.9433	0.0054
2	2	0	2	1	1	3992.0462	0.0021

J	K_a	K_c	J'	K'_a	K'_c	**Observed Freq. (MHz)**	**Residuals (MHz)**
7	2	6	7	1	7	4473.0647	-0.0044

Table S7: List of assigned rotational transitions of ^{13}C2 diphenylsilane in the 2-8 GHz frequency range.

J	K_a	K_c	J'	K'_a	K'_c	**Observed Freq. (MHz)**	**Residuals (MHz)**
2	1	2	1	0	1	2687.8060	0.0018
2	2	1	1	1	0	5393.8840	-0.0024
3	1	3	2	0	2	3339.9839	-0.0020
3	2	1	2	1	2	6156.2619	-0.0106
4	1	4	3	0	3	3977.6455	-0.0023
4	2	2	3	1	3	6905.8252	-0.0005
4	2	2	4	1	3	3867.9614	-0.0125
5	0	5	4	1	4	2289.9198	0.0058
5	1	5	4	0	4	4601.8700	-0.0029
5	2	4	5	1	5	4245.8867	0.0006
6	0	6	5	1	5	3048.8763	0.0026
7	0	7	6	1	6	3814.2174	-0.0069
8	1	8	7	0	7	6410.6660	0.0057
3	2	2	2	1	1	6061.2657	0.0093
4	2	3	3	1	2	6713.1450	-0.0059
5	2	4	4	1	3	7349.6288	0.0036
6	2	4	6	1	5	3730.8664	0.0028
5	2	3	5	1	4	3802.7784	-0.0067
2	2	0	2	1	1	3967.3048	-0.0010

J	K_a	K_c	J'	K'_a	K'_c	**Observed Freq. (MHz)**	**Residuals (MHz)**
2	2	1	2	1	2	4059.1355	0.0009
3	2	2	3	1	3	4105.6361	-0.0042
4	2	3	4	1	4	4167.8415	0.0004
6	2	5	6	1	6	4339.9418	0.0009
3	3	0	3	2	1	6685.8613	-0.0019
4	3	2	4	2	3	6689.9623	-0.0017
5	3	3	5	2	4	6692.9056	0.0042
6	3	4	6	2	5	6698.0513	0.0076
7	3	5	7	2	6	6706.2295	0.0074

Table S8: List of assigned rotational transitions of ^{13}C3 diphenylsilane in the 2-8 GHz frequency range.

J	K_a	K_c	J'	K'_a	K'_c	**Observed Freq. (MHz)**	**Residuals (MHz)**
2	2	0	1	1	1	5451.9698	-0.0008
2	2	1	1	1	0	5420.9345	-0.0054
3	1	3	2	0	2	3342.5264	0.0063
3	2	1	2	1	2	6179.5920	-0.0093
3	2	2	2	1	1	6085.4752	0.0004
4	1	4	3	0	3	3977.5891	-0.0037
4	2	2	3	1	3	6925.5070	0.0061
5	1	5	4	0	4	4599.3149	0.0005
5	2	3	5	1	4	3837.4379	-0.0011
6	0	6	5	1	5	3017.9723	-0.0020
6	1	6	5	0	5	5209.1250	-0.0090
2	1	2	1	0	1	2693.0369	-0.0050
8	1	8	7	0	7	6400.6934	0.0086
4	2	3	3	1	2	6734.6635	-0.0096
7	1	7	6	0	6	5808.8818	0.0078
6	2	4	6	1	5	3765.8136	-0.0018
4	2	2	4	1	3	3902.2432	-0.0031
3	2	2	3	1	3	4137.9348	-0.0100
4	2	3	4	1	4	4199.5786	-0.0036

J	K_a	K_c	J'	K'_a	K'_c	**Observed Freq. (MHz)**	**Residuals (MHz)**
5	2	4	5	1	5	4276.9110	-0.0049
12	2	11	12	1	12	5270.0863	0.0036
4	3	2	4	2	3	6745.1462	0.0040
5	3	3	5	2	4	6748.0082	0.0035
6	3	4	6	2	5	6753.0196	0.0028
7	3	5	7	2	6	6761.0021	0.0120

Table S9: List of assigned rotational transitions of ^{13}C4 diphenylsilane in the 2-8 GHz frequency range.

J	K_a	K_c	J'	K'_a	K'_c	**Observed Freq. (MHz)**	**Residuals (MHz)**
2	2	0	1	1	1	5464.6209	0.0011
2	2	1	1	1	0	5433.9485	-0.0035
3	2	1	2	1	2	6192.1629	-0.0054
4	1	4	3	0	3	3984.6519	-0.0042
4	2	3	4	1	4	4210.0074	0.0008
5	2	3	4	1	4	7703.3406	0.0028
5	2	4	4	1	3	7384.1509	0.0115
6	1	6	5	0	5	5218.5300	0.0089
6	2	5	6	1	6	4378.5496	0.0068
8	1	8	7	0	7	6412.5681	0.0097
2	1	2	1	0	1	2698.2712	-0.0017
3	1	3	2	0	2	3348.5923	0.0014
7	1	7	6	0	6	5819.5123	0.0030
3	2	2	2	1	1	6099.1541	-0.0029
4	2	3	3	1	2	6749.1970	-0.0052
9	1	9	8	0	8	6999.9998	-0.0132
7	3	5	7	2	6	6780.8650	-0.0065
6	3	4	6	2	5	6773.1027	0.0039
5	3	2	5	2	3	6750.7643	0.0041

J	K_a	K_c	J'	K'_a	K'_c	**Observed Freq. (MHz)**	**Residuals (MHz)**
7	2	6	7	1	7	4486.4795	0.0008
3	2	1	3	1	2	3970.6251	-0.0046
4	2	2	4	1	3	3915.9683	0.0027
5	2	3	5	1	4	3851.7382	0.0008
4	2	2	3	1	3	6937.7242	-0.0096

Table S10: List of assigned rotational transitions of ^{13}C5 diphenylsilane in the 2-8 GHz frequency range.

J	K_a	K_c	J'	K'_a	K'_c	**Observed Freq. (MHz)**	**Residuals (MHz)**
2	2	1	1	1	0	5422.9052	-0.0004
3	2	1	2	1	2	6185.5133	0.0039
3	2	1	3	1	2	3951.7325	0.0054
3	2	2	2	1	1	6091.1067	0.0010
4	1	4	3	0	3	3990.3435	-0.0010
4	2	2	3	1	3	6935.3310	0.0051
4	2	3	4	1	4	4194.5228	-0.0035
5	1	5	4	0	4	4615.6228	0.0000
5	2	3	4	1	4	7705.5644	0.0003
5	2	4	4	1	3	7381.4216	0.0028
6	1	6	5	0	5	5228.9740	-0.0055
7	1	7	6	0	6	5832.2554	0.0057
3	1	3	2	0	2	3351.6817	-0.0040
2	1	2	1	0	1	2698.5801	-0.0039
4	2	3	3	1	2	6743.9269	0.0015
8	0	8	7	1	7	4578.4421	0.0031
7	0	7	6	1	6	3808.6260	-0.0058
6	0	6	5	1	5	3042.7319	-0.0039
7	3	5	7	2	6	6751.9557	0.0010

J	K_a	K_c	J'	K'_a	K'_c	**Observed Freq. (MHz)**	**Residuals (MHz)**
4	3	1	4	2	2	6728.2082	-0.0180
6	2	5	6	1	6	4365.5464	0.0008
3	2	2	3	1	3	4132.7223	0.0103
2	2	1	2	1	2	4086.4988	0.0052
4	2	2	4	1	3	3896.3743	-0.0073
6	2	4	6	1	5	3759.6998	0.0040

Table S11: List of assigned rotational transitions of ^{13}C6 diphenylsilane in the 2-8 GHz frequency range.

J	K_a	K_c	J'	K'_a	K'_c	**Observed Freq. (MHz)**	**Residuals (MHz)**
2	2	0	1	1	1	5471.1873	-0.0062
2	2	1	1	1	0	5439.8186	-0.0035
3	1	3	2	0	2	3362.5222	-0.0025
3	2	1	2	1	2	6205.4626	-0.0089
3	2	2	2	1	1	6110.3195	0.0175
4	1	4	3	0	3	4003.2295	-0.0038
4	2	2	3	1	3	6958.2405	-0.0037
5	0	5	4	1	4	2292.9269	0.0034
5	1	5	4	0	4	4630.4696	0.0052
6	1	6	5	0	5	5245.6971	0.0010
7	0	7	6	1	6	3824.1273	0.0047
7	1	7	6	0	6	5850.7911	0.0042
2	1	2	1	0	1	2707.2604	-0.0023
8	1	8	7	0	7	6447.9191	-0.0092
4	2	3	3	1	2	6765.2807	0.0020
2	1	2	1	0	1	2707.2604	-0.0023
6	0	6	5	1	5	3055.2872	-0.0008
6	3	3	6	2	4	6727.8001	0.0292
7	3	4	7	2	5	6707.5292	-0.0102

J	K_a	K_c	J'	K'_a	K'_c	**Observed Freq. (MHz)**	**Residuals (MHz)**
6	2	5	6	1	6	4380.1415	-0.0032
3	2	2	3	1	3	4145.4339	-0.0050
3	2	1	3	1	2	3963.0126	-0.0117
4	2	2	4	1	3	3907.2606	-0.0065
6	2	4	6	1	5	3769.6553	0.0057
4	3	1	4	2	2	6748.1316	-0.0015

S5.0 Comparison of barrier heights at different levels of theory.

Table S12: Tunnelling barrier heights derived from single-point energies calculated for the equilibrium structure (ES) and transition state structure (TS) in cm^{-1} on the geometries optimized at B3LYP-D3/aug-cc-pVTZ level of theory.

Method	Barrier	DPS (cm^{-1})	DPE (cm^{-1})
DLPNO-CCSD(T)	TS-ES	82	57
CCSD(T)	TS-ES	90	51
CC2	TS-ES	108	27
RI-MP2	TS-ES	73	69
